# The Spectrum of Sleep Disorders Among Children: A Cross-sectional Study at a South Indian Tertiary Care Hospital

**DOI:** 10.7759/cureus.7535

**Published:** 2020-04-04

**Authors:** Udayakumar Narasimhan, Fatima Shirly Anitha, Chamelee Anbu, Mohammed Fazil Abdul hameed

**Affiliations:** 1 Pediatrics, Sri Ramachandra Institute of Higher Education and Research, Chennai, IND; 2 Psychiatry, Saveetha Medical College and Hospital, Chennai, IND; 3 Pediatrics, King Fahad Military Medical Complex, Dhahran, SAU

**Keywords:** pediatric sleep disorders, sleep hyperhidrosis, sleep problems

## Abstract

Introduction

Sleep problems during infancy and early childhood are fairly common and rarely recognized in pediatric practice. These are mostly related to the initiation and maintenance of night-time sleep. Understanding sleep patterns and disorders associated with sleep is challenging, especially in the pediatric age group. This study was done to estimate the magnitude of sleep disorders in children and to evaluate the associated risk factors.

Methods

This cross-sectional study was carried out among 450 children visiting the pediatric outpatient department of Sri Ramachandra Institute of Higher Education and Research, Chennai, India between November 2018 and June 2019. Children with chronic illnesses and a history of physical or mental trauma in the past six months were excluded. The Sleep Disturbance Scale for Children (SDSC) was used to gather information regarding sleep disorders.

Results

It was observed that a majority of the participants (72.2%) slept 9-11 hours per day. Among 46.2% of the participants, the time lag between bedtime and sleep time was less than 15 minutes. Overall, sleep problems were present in 34% of the participants. History of sleep problems in infancy, absence of siblings, and parental presence while sleeping emerged as statistically significant risk factors for childhood sleep disorders (p: <0.05).

Conclusion

We believe our study provides a basis for exploring the pattern and problems associated with sleep behavior among children. There is a need for setting up routine screening measures in pediatric outpatient departments to facilitate early detection of sleep disorders in order to avoid complications.

## Introduction

Sleep is an essential component of human biology and any disturbance in sleep affects the cognitive, emotional, and physical well-being of the individual. Diagnosis and treatment of sleep disorders are challenging, especially in the pediatric population. Sleep problems during infancy and early childhood are usually related to the initiation and maintenance of sleep [1]. The average sleep requirement of a child ranges from 16 to 18 hours during the first year of life and gradually decreases to 10 hours per night during childhood and adolescence [2].

Several studies have documented the prevalence of sleep disturbances among school children to be 35-46% [3,4]. Extensive research has been carried out in the field of sleep medicine in the past few decades. Measures were taken to standardize and quantitatively assess various disorders of sleep disturbance, leading to the development of the Sleep Disturbance Scale for Children (SDSC) [5]. Based on this scale, researchers were able to quantify the magnitude of sleep disorder in general and also assess six individual sleep disorders: disorders of initiating and maintaining sleep (DIMS), sleep breathing disorders, disorders of arousal, sleep-wake transition disorders, disorders of excessive somnolence, and sleep hyperhidrosis (SHY).

The prevalence of these individual disorders varies among different population groups. Among Indian children, the prevalence of individual sleep disorders is 3.2-25.5% [6]. The magnitude of this problem is indirectly represented in Indian studies and is associated with complaints like bed wetting, sleep talking, sleepwalking, teeth grinding, and night terrors. It is crucial to detect and identify these symptoms at an early stage in children in order to prevent physical and behavioral deterioration.

## Materials and methods

Our study aimed to estimate the prevalence and risk factors of sleep disorders among Indian school children. This cross-sectional study was carried out between November 2018 and June 2019 in the outpatient department of pediatrics at Sri Ramachandra Institute of Higher Education and Research, Chennai, India.

Based on previous studies, the lowest prevalence of sleep disorders in India was found to be 3.2% [6]. At a 95% level of significance and 1.75% absolute precision, the sample size was estimated to be 388.4. Factoring in 10% of incomplete responses, the sample size was calculated as 427.2 and was rounded off to 450. The participants were selected by convenient sampling based on the selection criteria. All patients who visited the outpatient clinic of the department of pediatrics were selected for the study. Based on the selection criteria, a total of 450 participants were enrolled. Normal and healthy children between 1-13 years of age were included in the study after obtaining informed consent from the parents. Children with chronic medical illnesses, those who had been prescribed drugs for any medical illness for over one year, and those with a history of physical injuries or mental illness as evaluated by a child psychiatrist during the previous six months were excluded from the study.

Approval was obtained from the Institutional Research Ethics Committee of Sri Ramachandra Institute of Higher Education and Research prior to the commencement of the study. Parents were informed in detail about the study, and informed consent was obtained from them prior to the commencement of data collection. A structured interview schedule was employed to obtain information from the parents of the participants. The interview schedule consisted of background information regarding age, gender, education level of parents, and socioeconomic status. The SDSC was used for obtaining information regarding sleep disorders in the past six months. The questionnaire based on the scale consisted of 26 questions related to sleep behaviors and abnormal sleeping patterns, the answers to which were graded based on the frequency of the symptom. The total score was computed and participants who had a total score of more than 39 were considered to have sleep problems [5]. Among the 26 questions, individual sleep disorders were assessed by summing the scores of certain groups of questions. This included DIMS, sleep breathing disorders, disorders of arousal, sleep-wake transition disorders, disorders of excessive somnolence, and SHY. This corresponds to the International Classification of Diseases 10th revision (ICD-10) diagnosis of sleep disorders such as circadian rhythm sleep disorders, sleep-related breathing disorders, and hypersomnia. For each question, when the frequency of the symptom was three or more times per week, it was considered a positive symptom. The parents of children diagnosed with sleep problems were educated about sleep hygiene measures.

Data was entered and analyzed using IBM SPSS software Version 17.0 (IBM, Armonk, NY). Mean scores were computed for the study participants. A chi-square test was used to analyze the association between sleep disorders and risk factors. The independent samples t-test was used to compare the mean scores with risk factors, and a p-value of <0.05 was considered statistically significant.

## Results

This study was carried out among 450 children at our tertiary care hospital. The majority of the participants were above five years of age and boys outnumbered girls (Table 1). The mean body mass index (BMI) of the study participants was 15 ±2.9 kg/m^2^, which lies between the 5th-50th percentile according to Indian standards.

**Table 1:** Demographic profile of the study participants

Demographic parameter	Number of patients (n = 450)	Percentage (%)
Age, years		
<5	221	49.1
5-10	190	42.2
>10	39	8.7
Gender		
Female	218	48.4
Male	232	51.6
Educational status of fathers		
Illiterate	43	9.6
School-level	93	20.7
Graduation	148	32.9
Professional	166	36.9
Educational status of mothers		
Illiterate	45	10.0
School-level	100	22.2
Graduation	154	34.2
Professional	151	33.6
Siblings		
Absent	189	42.0
Present	261	58.0

In the present study, a family history of sleep disorders was present in 3.8% of the participants, and sleep disturbance was found to be the predominant sleep disorder, affecting 76.5% of families in the study population (Table 2).

**Table 2:** Sleep history of the study participants

Characteristics	Number of patients (n = 450)	Percentage (%)
Sleep problems in infancy		
Present	32	7.1
Absent	418	92.9
Family history of sleep disorders		
Present	17	3.8
Absent	433	96.2
Family member suffering from sleep disorders (n = 17)		
Father	8	47.0
Grandfather	5	29.4
Grandmother	2	11.8
Mother	2	11.8

The SDSC was used to analyze the quality and quantity of sleep in our study, and it revealed that the majority of the participants (72.2%) slept for 9-11 hours per day. For 46.2% of the participants, the time lag between bedtime and sleep time was less than 15 minutes. Sleep disturbances were present in more than one-third (34%) of the study participants. About 7.3% of the participants were always reluctant to fall asleep and 4.2% of the participants had sudden jerks during sleep almost every day; 7.1% occasionally experienced excessive sweating during sleep. Occasional snoring was present in 9.1% of the participants while occasional sleepwalking was present in 4.7% of the participants. Moreover, about 10.7% of the participants found difficulty in waking up in the morning on all days.

It was observed that the presence of sleep problems during infancy is a significant risk factor for sleep disorders in childhood (p: 0.002). Children with sleep disorders were found to be 1.6 times more likely to be without any sibling in the present study and association was found to be statistically significant (p: 0.007) (Table 3).

**Table 3:** Association between sleep disorders and various risk factors *Chi-square test

Sleep disorder (SDSC score >39)			Number of patients (n = 450)					Percentage (%)	
Present			153					34.0	
Absent			297					66.0	
Risk Factor	Number of patients (n = 450)	Sleep disorder			Odds ratio	95% confidence interval			P-value
		Present, n (%)		Absent, n (%)					
Sleep problems in infancy									
Present	32	20 (62.5)		12 (37.5)	3.5		1.6-7.5		0.002*
Absent	418	133 (31.9)		285 (68.1)					
Siblings									
Absent	189	77 (40.7)		112 (59.3)	1.6		1.1-2.4		0.007*
Present	261	76 (29.1)		185 (70.9)					
Family history of sleep disorders									
Present	17	10 (58.8)		7 (41.2)	2.8		1.0-7.7		0.029*
Absent	433	143 (33.0)		290 (67.0)					
Parental presence while sleeping									
Present	324	121 (37.3)		203 (62.7)	1.2		1.1-2.7		0.016*
Absent	126	32 (25.4)		94 (74.6)					

## Discussion

The presence of sleep disorders indicates subtle alterations in the neurophysiology of the child and causes behavioral changes in children. On average, the majority of the participants in the present study slept 9-11 hours per day. A study by Rosen et al. has reported an average sleep duration of 7.5 hours, which is lower than in our study [7]. The longer sleep duration in our study can be explained by parental influences, which stem from the common practice of kids sleeping with parents in India.

In our study, the overall prevalence of sleep disorders was 34%, with SHY being the most common sleep-specific disorder. An Indian study by Gupta et al. found that daytime napping was the most common disorder (56%) [6]. However, this study included only children above five years of age and sleep was assessed using children’s sleep habit questionnaire (CSHQ). Schmid et al. conducted a study to identify whether regulatory problems at five months of age were predictive of future sleep and social problems. The prevalence of sleep disorders at 20 months of age was 17.8%, and it was 15.5% at 56 months of age. In 8% of infants, regulatory problems like feeding and sleeping difficulties persisted well into preschool years [8]. Another study done by Fricke-Oerkermann et al. reported the prevalence of sleep disorders to be 30-40% among children, which is also similar to our findings [9].

Our study also observed that parental education and socioeconomic status significantly influence the occurrence of sleep disorders. Sleep patterns in children can be influenced by parental education, employment, and socioeconomic status. Nonstandard irregular work patterns among parents were found to have negative influences on the child’s cognitive outcome and sleep, which was prevalent more in single-parent and low-income families. Children who hail from low socioeconomic backgrounds compounded by family stress, maternal anxiety, and depression are at risk of specific sleep problems [10]. A study done in China by Jiang et al. confirmed that mobile phone use is linked to difficulty in maintaining sleep among children [11]. This can have a significant negative effect on school performance, and delaying school start timings in the morning has been proposed in one study [12].

Our study revealed several interesting findings. One of the key features of our study is the assessment of the impact of having no siblings on the prevalence of sleep disorders in children. Our study demonstrated that not having siblings is a significant risk factor for two specific sleep disorders, namely DIMS and SHY. This is an indirect indicator of the prevalence of room-sharing among siblings. In our study, parental presence during sleep was associated with an increased risk of sleep disorders (p: 0.016). Room-sharing in infancy has been found to be associated with increased risk of requiring bed-sharing among children of preschool age in studies conducted in the west. Room-sharing has been linked to shorter sleep stretches, less sleep, and unsafe sleep practices, which increase the risk of sleep-related deaths [13]. However, an Indian study by Mishra et al. concludes that bed-sharing and sleeping with parents significantly improved the quality and duration of sleep among children and adolescents [14]. It was found that doctors suggested sleep evaluation for only seven children in our study population. This constituted about 4.5% of the 153 children diagnosed with sleep problems. This indicates that sleep problems are underestimated in routine pediatric practice and emphasizes the need for pediatricians to be sensitized in diagnosing sleep problems. One of the main limitations of our study was that we did not assess how children’s sleep was affected by the nicotine-consumption behavior of the parents, which is rare among females in our population.

## Conclusions

We believe that our study provides a basis for exploring the pattern and problems associated with sleep behavior among Indian children. Although the magnitude of specific sleep disorders found in our study is negligible, almost one-third of the study participants had sleep disturbance. The cultural milieu in India facilitates and promotes co-sleeping, which can be both beneficial and detrimental. Apart from a lack of awareness about sleep disorders, poor parental recall has also been reported as a key reason for the under-diagnosis of the condition in children. There is an urgent need for setting up routine screening measures in pediatric outpatient departments to facilitate better and early detection of sleep disorders in children. Educating parents and teachers to identify children with sleep disorders is essential and must be implemented as part of long-term healthcare plans in the country.
